# Diagnosis of Cystic Echinococcosis, Central Peruvian Highlands

**DOI:** 10.3201/eid1402.061101

**Published:** 2008-02

**Authors:** Cesar M. Gavidia, Armando E. Gonzalez, Wenbao Zhang, Donald P. McManus, Luis Lopera, Berenice Ninaquispe, Hector H. Garcia, Silvia Rodríguez, Manuela Verastegui, Carmen Calderon, William K.Y. Pan, Robert H. Gilman

**Affiliations:** * Universidad Nacional Mayor de San Marcos, San Borja, Lima, Peru; † The Queensland Institute of Medical Research, Brisbane, Queensland, Australia; ‡ Universidad Peruana Cayetano Heredia, Lima, Peru; § Instituto de Ciencias Neurologicas, Lima, Peru; ¶ Johns Hopkins University, Baltimore, Maryland, USA

**Keywords:** Cystic echinococcosis, diagnostic-test prevalence, IBCF, rEpC1-GST, ultrasound, X-ray, Peru, research

## Abstract

We evaluated prevalence of cystic echinococcosis (CE) in a central Peruvian Highland district by using 4 diagnostic methods: ultrasonography for 949 persons, radiography for 829, and 2 serologic tests for 929 (2 immunoblot formats using bovine hydatid cyst fluid [IBCF] and recombinant EpC1 glutathione S-transferase [rEpC1-GST] antigens). For the IBCF and rEpC1-GST testing, prevalence of liver and pulmonary CE was 4.7% and 1.1% and seropositivity was 8.9% and 19.7%, respectively. Frequency of seropositive results for IBCF and rEpC1-GST testing was 35.7% and 16.7% (all hepatic cysts), 47.1% and 29.4% (hepatic calcifications excluded), and 22.2% and 33.3% (lung cysts), respectively. Weak immune response against lung cysts, calcified cysts, small cysts, and cysts in sites other than lung and liver might explain the poor performance of the serodiagnostic tests. We confirm that CE is highly endemic to Peru and emphasize the limited performance of available serologic assays in the field.

## Abstract 2

High prevalence was confirmed by ultrasonography, radiography, and 2 serologic tests, although usefulness of serologic testing in the field was limited.

Cystic echinococcosis (CE), caused by the larval stage of *Echinococcus granulosus*, is recognized as a public health problem (*1*). Cysts develop in internal organs of intermediate hosts (herbivores and humans). The disease represents a challenge of increasing concern in countries where control programs have been reduced or have not yet been implemented (*2*,*3*). Globally, the annual loss due to human hydatidosis (treatment and lost income) has been estimated at ≈US $200 million (*4*).

CE is endemic to >100 countries in Latin America, Asia, and Africa (*1*,*5*) and is considered an emerging disease in other areas. In the former Soviet Union and Eastern Europe, the number of cases has dramatically increased in recent years (*6*–*8*). The annual incidence of CE hospital cases has reached >8/100,000 persons in some European countries, and 42/100,000 in Xinjiang, People’s Republic of China (*5*). The highest incidence of surgical cases (198/100,000) has been reported in Kenya (*1*). A few areas (Iceland, Ireland, and Greenland) are believed to be free of autochthonous transmission. The United States has reported a few cases in livestock; most CE infections in persons are imported. This is also true for regions of western and central Europe (*4*) with the exception of countries such as Spain, where the parasite is prevalent and remains a major public health problem (*9*,*10*).

Studies in Peru have shown high prevalence of CE in humans, particularly in the central and southern highlands (*11*,*12*). During 1997–1999, prevalence in the central Andes was 5.7%–9.3% according to ultrasonography, radiography, or both and up to 18.2% according to immunoblot testing (*11*,*12*). Portable ultrasonography has facilitated the study and more accurate reporting of CE prevalence in endemic regions (*13*,*14*), following the standardized World Health Organization classification (*15*).

Among available serologic tests, the immunoblot (IB) assay that uses bovine hydatid cyst fluid (IBCF) has been successfully used in CE-endemic areas of Peru (*12*,*14*). This IBCF has a sensitivity of 80% for hepatic cysts and 56% for pulmonary cysts (*16*). Another immunoblot test, which uses a purified recombinant EpC1 glutathione S-transferase antigen (rEpC1-GST), has a sensitivity of 92.2% and a specificity of 95.6% (*17*). An immunoreactive clone (*EpC1),* encoding EpC1 was identified by immunoscreening a cDNA library constructed with RNA extracted from protoscolices from sheep hydatid cysts. Immunoglobulin (Ig) G was the dominant antibody isotype generated against rEpC1-GST (*17*). Hitherto, no field testing of the EpC1-GST had been undertaken. Measuring the real extent of CE in South America as well as evaluating the native, recombinant, and peptide antigens for diagnosis of CE in humans have been recommended (*18*). During July and August 2004, we used 4 diagnostic methods—ultrasonography, chest radiography, and 2 serologic assays—to evaluate the prevalence of CE in humans in an unexplored CE-endemic area of the central Peruvian Highlands where control measures have been attempted incompletely.

## Materials and Methods

### Study Site

**Figure 1:**
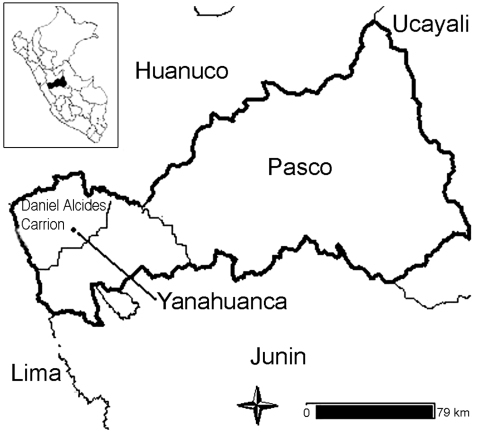
Map of the Central Peruvian Highlands.

We selected 9 rural communities located 5–50 km from Yanahuanca district, which is located in the Pasco department (central Peruvian Andes) at 3,249–4,314 m above sea level (Figure 1). The terrain is mountainous and roads are unpaved. Houses are made of adobe, and drinking water is obtained from streams or rivers. Primary healthcare is provided by health centers; specialized care is available from the closest hospital in Pasco (40–60 km).

Sheep (n = 99,175) are the dominant livestock, but cattle (n = 5,451), swine (n = 2,784), alpacas (n = 2104), llamas (n = 5,679), and guinea pigs (n = 8,870) (*19*) are raised for human consumption. With the exception of guinea pigs, animals are kept in fields distant from the villages. Dogs are routinely used as shepherds, but some are kept as pets.

### Study Design

After coordination with local authorities, a census was taken of persons in each village; to maintain confidentiality, persons were assigned a code. A cross-sectional study was performed by using ultrasonography of the abdomen, radiography of the chest, and 2 immunoblot assays with different antigens (crude IBCF [*16*] and a recombinant antigen rEpC1-GST [*17*]). Persons >5 years of age were invited to participate. All examinations were conducted at community health centers; 3–4 mL of blood was taken from all persons who volunteered to participate in the study. Women of childbearing age were asked to have a urine pregnancy test, and those who were pregnant were excluded from radiographic examination. The ethics review boards of the Universidad Peruana Cayetano Heredia and the Bloomberg School of Public Health of Johns Hopkins University approved the study and written consent forms.

### Radiography

Posterior-anterior portable radiographs were taken by using a Polyskop machine (Siemens, Orlando, FL, USA). A radiologist, who was not provided serologic and ultrasonography results, read the films and classified the findings by using the Beggs criteria for lung CE (*20*). Unruptured cysts were defined as centrally located, closed, well-defined, and round lesions.

### Ultrasonography

Ultrasonography was performed with a portable 3.5-MHz ultrasonograph (model Shimasonic SLD-32, Shimatzu, Kyoto, Japan). Cysts were diagnosed by using the international classification of ultrasound images for CE, which classifies cysts in cystic lesions, and from CE type 1 to CE type 5 according to their development grade or degeneration (*15*).

### IBCF Immunoblot

Bovine hydatid cysts were obtained from abattoirs, and fluid was aspirated and placed in a beaker at 4°C to which 0.5 M phenylmethylsulfonyl fluoride (Sigma, St. Louis, MO, USA) was added (1:100 dilution). After centrifugation at 3,000 *g* for 10 min, the supernatant was lyophilized and stored at –20°C until use. The antigen was diluted with 0.1% sodium dodecyl sulfate (SDS), 0.025 (w/v) bromophenol blue, 0.0025 M Tris-HCl (pH 8.0), and the dilution was completed with 6% glycerol to give a final concentration of 0.2 μg/μL. The antigen was resolved by polyacrylamide gel electrophoresis as described elsewhere (*16*,*21*). The separated proteins were electrotransferred to nitrocellulose membrane, cut into strips, and immersed in a dilution of 1:25 (serum:phosphate-buffered saline with 0.3% Tween 20). Bound human antibodies were detected by incubating each strip in horseradish peroxidase–conjugated goat antibody to human IgG at a dilution of 1:1,000. Antibodies bound to diagnostic bands of 8, 16, and 21 kDa were seen after addition of 3,3′-diaminobenzidine. A positive result was defined as the presence of any diagnostic band (*16*).

### rEpC1-GST Immunoblot

rEpC1-GST was expressed in *Escherichia coli* (*21*). The rEpC1-GST fusion protein was subjected to electrophoresis on 12% (w/v) SDS-polyacrylamide gels under reducing conditions and then transferred onto nitrocellulose membrane; the membrane was then cut into strips containing ≈0.3 μg of rEpC1-GST protein, as described (*17*). After being blotted with 5% (w/v) skim milk, the strips were incubated with human serum samples (diluted 1:100) for 1 h at 37°C and washed 3× with phosphate-buffered saline Tween before being incubated with goat anti-human whole immunoglobulin IgG conjugate (Sigma). After development in 4-chloro-1-naphanol substrate solution for 15 min at room temperature, the strips were examined. A positive serum sample showed a band of ≈41 kDa (*17*).

### Data Analysis

The prevalence of CE was determined for the 2 imaging techniques, and the proportion of seropositive persons was estimated according to serologic test results. The test prevalence was calculated for all communities, and the difference was assessed by a 2-sample test of proportions. The χ^2^ test was used to evaluate the association of sex with positive results for all 4 tests. The frequency of seropositivity (by IBCF or rEpC1-GST) for persons who had CE-positive ultrasonographic or radiographic images was calculated to evaluate the performance of the immunoblot tests. This frequency was, moreover, assessed with and without hepatic calcified cysts, and for liver cysts <20 mm (24 persons) or >20 mm (18 persons) in diameter. The κ test was used to establish the agreement between the 2 serologic assays and with either ultrasonography or radiography. All statistical analyses were computed by using Stata 8.0 (Stata Corporation, College Station, TX, USA) with a significance level of <0.05.

## Results

Of the 1,973 persons registered during the census, 137 (7%) were <5 years of age, which left 1,836 potential study participants. Of these, 949 persons (51.7%) were examined. All 949 were evaluated with ultrasonography, 829 had chest radiographs taken, and 929 contributed blood samples. The proportion of females was higher among participants (60.3%) than nonparticipants (50%) (p<0.05). Of those with ultrasonographic results, 39 had no serologic results because they refused to have their blood collected. In addition, 125 persons had serologic results but refused to have chest radiographs taken. The ages of participants and community members were similar (mean age 28.8 and 28 years, respectively) when children <5 years of age were excluded from analysis.

Ultrasonography showed prevalence of CE in the liver to be 4.7% (45/949); radiography showed prevalence of CE in the chest to be 1.1% (9/829). Two persons had cysts in the liver and lungs. Therefore, the CE prevalence according to ultrasonography and radiography was 5.5% (52/949; 95% confidence interval [CI] 4.1%–7.1%). Seropositivity according to IBCF was 8.9% (83/929; 95% CI 7.2%–10.9%); seropositivity according to rEpC1-GST was significantly higher at 19.7% (184/929; 95% CI 17.2%–22.4%, p<0.01). All 83 IBCF-positive persons reacted to the 16-kDa band; the bands of 21 and 8 kDa were observed for 78 and 76 persons, respectively. No differences were found among the 9 communities (Table 1). When participants were divided into 2 groups according to median age (<23 and >23 years), no difference was found for proportion of those who were positive by serologic assay and by radiographic examination; however, a significant difference existed according to ultrasonographic examination (14/456 [3.1%] for those <23 years vs. 31/493 [6.3%] for those >23 years; p<0.05).

**Table 1:** Immunoblot assay results for cystic echinococcosis, central Peruvian Highlands,  using 2 immunoblot assays with different antigens*

Community	No. samples	Antigen, no. (%) positive	
		IBCF	rEpC1-GST
Tambochaca	62	2 (3.2)	17 (27.4)
Huarautambo	54	2 (3.7)	10 (18.5)
Astobamba	79	5 (6.3)	12 (15.2)
Santiago Pampa	213	17 (8)	35 (16.4)
12 de Octubre	84	11 (13.1)	10 (11.9)
Andachaca	118	11 (9.3)	25 (21.2)
Uchumarca	113	15 (13.3)	27 (23.9)
Tambopampa	107	10 (9.4)	30 (28)
Ayayog	74	6 (8.1)	14 (18.9)
Other†	25	4 (16)	3 (12)
Total	929	83 (8.9)‡	183 (19.7)‡

*IBCF, antigen was bovine hydatid cyst fluid; rEpC1-GST, antigen was recombinant EpC1 glutathione S-transferase. †Group of volunteers who came from nearby communities and were also included in the survey. ‡Statistically different (p<0.01), with higher percentage of seropositivity for rEpC1-GST than IBCF.

**Figure 2:**
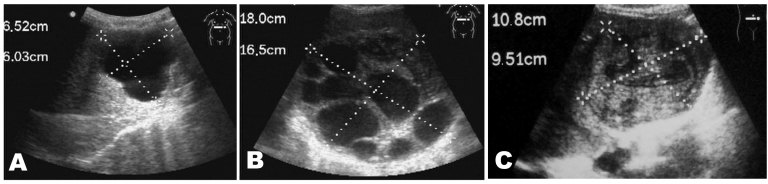
Ultrasonographic images of cystic echinococcosis in the liver in patients from the Yanahuanca district, Central Peruvian Highlands. A) Cyst type CE1; B) Cyst type CE2; C) Cyst type CE4.

The total number of liver cysts was 50 (5 persons had 2 liver cysts each); the total number of lung cysts was 10 in 9 persons. The lung-to-liver ratio was 1:5. Most of the liver cysts were classified as CE5 (54%, 27/50), inactive cysts with calcified walls, followed by CE1 (20%, 10/50) active cysts and CE2 (10%, 5/50). Types CE3 and CE4 with signs of initial degeneration (8% each, 4/50) were rare (Figure 2). The average age was similar for persons with CE1, CE4, and CE5 at 40.6, 40.9, and 40 years of age, respectively. Those with CE2 and CE3 averaged 28.8 and 23.3 years of age, respectively.

The frequency of persons in the group that had CE-positive imaging results who also had IBCF-positive results was 47.1% (8/17; 95% CI 23%–72.2%) for hepatic noncalcified cysts, 35.7% (15/42; 95% CI 21.6%–52%) when hepatic calcified cysts were included, and 22.2% (2/9; 95% CI 2.8%–60%) for pulmonary cysts. Of the 52 persons who had CE in liver, lungs, or both, 49 provided serum samples (3 who had positive ultrasonography results did not provide a blood sample). The frequency of persons in the group that had CE-positive imaging results who also had rEpC1-GST–positive results was 16.7% (7/42; 95% CI 7%–31.3%) for all hepatic cysts, and 29.4% (5/17; 95% CI 10.3%–56%) when hepatic calcified cysts were excluded. This test detected 33.3% (3/9; 95% CI 7.5%–70%) of those with CE-positive chest radiographs. Neither serologic test detected >50% of persons with presumptive CE imaging results (Table 2). However, the IBCF was >2× as sensitive as the rEpC1 for detecting persons with CE-positive imaging results.

**Table 2:** Frequency of seropositive results among persons with cystic echinococcosis–positive ultrasonography and radiography imaging results, central Peruvian Highlands*

Antigen	Ultrasonography,† % (95% CI)		Radiography‡	Overall,§ % (95% CI)	
	With calcification	Without calcification		With calcification	Without calcification
IBCF	35.7 (21.6–52)	47.1 (23–72.2)	22.2 (2.8–60)	32.7 (20–47.5)	34.6 (17.2–55.7)
rEpC1-GST	16.7 (7–31.3)	29.4 (10.3–56)	33.3 (7.5–70)	18.4 (8.8–32)	26.9 (11.6–47.8)

*CI, confidence interval; IBCF, antigen was bovine hydatid cyst fluid; rEpC1-GST, antigen was recombinant EpC1 glutathione S-transferase. †Frequency of positive abdominal ultrasonographic results. ‡Frequency of positive chest radiographic results. §Ultrasonography, radiography, or both.

Of the 2 persons who had cysts in lung and liver, 1 had positive results for both serologic tests and the other had negative results for both. The frequency of seropositivity for persons with liver cysts <20 mm in diameter was 25% (6/24) for IBCF and 4% (1/24) for rEpC1-GST. In contrast, the frequency for persons with liver cysts >20 mm in diameter was 50% (9/18) for IBCF and 33% (6/18) for rEpC1-GST. A significant difference in detecting cysts with diameters <20 mm versus >20 mm was found for only rEpC1-GST (4%– 33%, p<0.05).

The agreement between IBCF and rEpC1-GST was only 8%, which would be expected by chance alone (κ = 0.08, p<0.01). Of 928 participants, 26 were positive by both serologic tests (2.8%), and 688 (74.1%) were negative by both. A total of 157 persons who had IBCF-negative results had rEpC1-GST–positive results; 57 who had IBCF-positive results had rEpC1-GST–negative results. CE-test positivity was not significantly associated with sex according to any diagnostic test (Table 3).

**Table 3:** Echinococcosis-positive results, by sex, 4 diagnostic tests, central Peruvian Highlands*

Test	Female			Male	
	No. (%) positive	Total		No. (%) positive	Total
Ultrasonography†	30 (5.2)	572		15 (4.0)	377
Radiography‡	7 (1.4)	497		2 (0.6)	332
IBCF§	54 (9.7)	558		29 (7.8)	371
rEpC1-GST¶	113 (20.3)	558		70 (18.8)	371

*By χ^2^ analysis, none of the tests showed a significant association between sexes with positive diagnosis. †Abdomen. ‡Chest. §IBCF, antigen was bovine hydatid cyst fluid. ¶rEpC1-GST, antigen was recombinant EpC1 glutathione S-transferase.

To assess possible cross-reactions with cysticercosis, we evaluated serum by using a purified glycoprotein *Taenia solium* antigen, in an immunoblot format (*18*). At the population level**,** antibodies to *T. solium* cysticercosis did not affect hydatid serologic assay results. The seroprevalence of cysticercosis was statistically similar for hydatid-seropositive and hydatid-seronegative persons, regardless of the antigen used to diagnose hydatidosis (IBCF: 11/83, 13.3% vs. 103/852, 12.1%, respectively; rEpC1-GST: 24/184, 13% vs. 90/750, 12%, respectively). Similarly, at the population level, the seroprevalence of *E.* *granulosus* antibodies was similar for cysticercosis-seropositive and cysticercosis-seronegative persons, regardless of the antigen used to detect *Echinococcus* (IBCF: 11/114, 9.7% vs. 72/821, 8.8%, respectively; rEpC1-GST: 24/114, 21.1% vs. 160/820, 19.5%, respectively).

## Discussion

This study demonstrates and confirms the high prevalence of CE in humans in the central Peruvian Highlands. It also highlights the limited performance of 2 immunoblot tests (IBCF and rEpC1-GST) under field conditions by detecting <50% of persons who had CE-positive imaging results. We show the utility of ultrasonography for CE screening, which demonstrated an elevated percentage (54%) of apparently inactive and calcified hepatic cysts.

The survey was well accepted by the study population. However, most of the participants were women; men tend to be more reluctant to participate in medical studies, especially those involving blood sampling. The CE prevalence of 5.5% found by ultrasonography and radiography is similar to previously reported rates from other central highland communities in Peru (4.9%–5.7%) (*12*,*14*). However, a study in Vichaycocha (north highland) showed a rate of 9.3% by ultrasonography and radiography, and a seropositivity rate of 18.2% (*11*). These rates are comparable to the highest reported prevalences in other countries such as China (liver CE from 3.3% to 6.6% [*22*,*23*]), Kenya (5.6% liver CE [*1*]), and Argentina before initiation of its control program (5.6% in school children [*24*]).

Unlike some other areas of Peru, Yanahuanca has not had a CE control program. This might explain, at least in part, why the overall prevalence in our survey was as high as 12.5% when ultrasonography, radiography, and IBCF results were combined and up to 23.8% when ultrasonography, radiography, and rEpC1-GST results were combined. Notwithstanding, these figures do not represent the true disease prevalence because they might reflect the continuous transmission and endemicity of *E. granulosus* in this region. Diagnostic approaches for CE based on imaging techniques can be problematic because of variations in size, shape, and location of the cysts. In addition, *E. granulosus* distribution, host susceptibility, and strain variation might affect disease transmission in different areas of the Peruvian Highlands (to our knowledge, no studies have tried to characterize *E. granulosus* strains in Peru).

One of the underlying weaknesses of this study was the lack of a true standard (a test with 100% sensitivity and 100% specificity), which would enable evaluation of alternative diagnostic tests and underlying prevalence. Most areas of medicine lack a true standard, yet recent statistical techniques have been developed that can help evaluate diagnostic tests and estimate true prevalence in the absence of such a standard. Most of these techniques rely upon a Bayesian framework (*25*–*27*) and are computationally intensive but more flexible than maximum likelihood–based approaches because they can incorporate correlation among diagnostic tests. Although the Bayesian approach offers distinct advantages, potential problems include specification of an appropriate prior distribution and a nonidentifiable model. Our study encountered both of these problems because reliable prior information is not readily available and estimates provided by a Bayesian approach are limited by the lack of identifiable groups.

The poor performances of the IBCF and rEpC1-GST testing may be related to false-positive imaging results from other space-occupying lesions (e.g., neoplasia, abscesses, nonparasitic cysts). Additionally, participants might have had low or undetectable levels of circulating antibodies from different stages of cyst development or degeneration. The production of IgG depends on the number, size, location, and condition of the cysts; only 60%–80% of persons with confirmed CE become seropositive (*28*). Calcified cysts are less seroreactive, thus decreasing seropositivity (*16*,*22*), as observed in this study. Previous studies using the same IBCF testing found that the frequency of seropositivity was 57% (*12*) and 53% (*14*) for liver hydatid cysts (ultrasonography), and 13% for lung cysts (*11*), similar to what we found in this study. However, another study, in which most of the liver cysts were active, reported a proportion of persons who were positive according to IBCF testing to be as high as 73% (*11*).

Other possible reasons for the limitation of serologic testing might be the weak immune response against pulmonary cysts, cysts at other sites (e.g., brain, eyes, bones, ovaries), small or poorly defined cysts, and a thick collagen cyst wall that would reduce antigen exposure (*29*). However, increased seropositivity (up to 50%) for detecting large hepatic cysts (>20 mm in diameter) has not been reported previously and may be due to elevated antigen concentrations in these cysts. To our knowledge, the only study showing a correlation between cyst size and seropositivity was performed in sheep (*30*).

The different antigen sources would explain the disparity and poor agreement between the 2 serologic tests. EpC1 is a recombinant antigen obtained from protoscolex larvae from sheep hydatid cysts (*17*), while the IBCF uses bovine hydatid cyst fluid (*16*). The IBCF appears to be more responsive than the rEpC1-GST in detecting CE-image cases. Crude hydatid fluid has been recommended for mass serologic screening (*31*) and purified antigen 5 (Ag5) and AgB for specific diagnosis. Ag5 and AgB are recognized as 2 of the most useful *E. granulosus* antigens for diagnosis (*32*), although Lorenzo et al. (*33*) found that hydatid cyst fluid, AgB, and its subunit AgB8/1 exhibited equivalent diagnostic efficiencies in a randomized multicenter study.

Among participants with CE-negative ultrasonography images, rEpC1-GST testing detected >3× more seropositive persons than the IBCF (19% vs. 7.3%, respectively). This scenario has been described with other diagnostic test such as AgB ELISA, which detected 5.3% CE-seropositive persons in a group with CE-negative ultrasonography images (*22*). We do not have evidence of cross-reaction with *Fasciola* for any of the serologic tests used in this study; however, *Hymenolepis nana, Entamoeba histolytica, Giardia lamblia,* and *Taenia sp* (*34*) are endemic to the study area, which might affect the serodiagnosis. We demonstrated no cross-reaction with cysticercosis (*T. solium*) by IBCF or rEpC1-GST because the proportion of persons who were cysticercosis positive was equal among those who were CE seropositive and CE seronegative according to both assays. Other possible explanations include past exposure to *Echinococcus* eggs (aborted infection) that produced only transient antibodies (*35*), and undetected cysts.

In our study, the lung-to-liver ratio of 1:5 is higher than ratios reported in other epidemiologic studies in Peru (*11*) but within the range of those reported in other *Echinococcus*-endemic South American countries such as Argentina, Chile, and Uruguay (1:3–1:13) (*11*,*36*). Molecular genetic studies of *E. granulosus* in the Peruvian Highlands may clarify some issues about the organ infection preferences (tropism), tissue survival, infection rates, immune responses, and the performance and agreement of immunodiagnostic tests.

Among liver cysts, 54% were CE5 and 20% were CE1; other studies have typically displayed an exponential decline in the frequency of liver cyst types from CE1 (most frequent) to CE5 (most rare) (*37*). Because most of these persons had not received a diagnosis of hydatid disease or antihelminthic treatment, these calcified forms are most probably the result of the natural process of degeneration driven by individual immune responses (*38*). A proportion of hydatid cysts die after initial establishment; thus, calcified lesions can be observed macroscopically (*29*). The geographic variation of cyst type frequencies can also depend on the time between infection and evaluation, the immune response of a highly exposed population, and *E*. *granulosus* genetic variation (*15*). Calcified cysts may also have been misdiagnosed with other lesions from biliary cysts, pyogenic abscesses, amebic liver abscesses, or even tumor-like masses or metastases (*39*), although these conditions are uncommon in Peru. We did not study the specific IgG subclasses in relation to cyst types, but their quantification may be important for understanding the natural history of hydatid cyst. IgG_4_ antibody response is associated with development, growth, and progression (CE1 to CE3); IgG_1_, IgG_2_, and IgG_3_ occur predominantly when cysts became infiltrated or degenerated (CE4 and CE5) (*40*).

Our study provides data on CE in the surveyed communities and shows the results of using ultrasonography, radiography, and immunodiagnosis for large-scale population screening. Determining baseline prevalence with ultrasonography enables the evaluation of epidemiologic surveillance activities and study of the natural history of CE. Ultrasonography is well accepted by the population and is relatively less expensive than other imaging techniques. Methods that are inexpensive and relatively easy to use, such as immunodiagnosis and ultrasonography, are required for large-scale screening of populations in which hydatidosis is endemic. However, serologic assays have serious limitations under field conditions, as has been demonstrated in this study. Seroepidemiologic surveys for CE require better diagnostic antigens and should be supported by imaging methods whenever possible.
